# Perovskite-molecule composite thin films for efficient and stable light-emitting diodes

**DOI:** 10.1038/s41467-020-14747-6

**Published:** 2020-02-14

**Authors:** Heyong Wang, Felix Utama Kosasih, Hongling Yu, Guanhaojie Zheng, Jiangbin Zhang, Galia Pozina, Yang Liu, Chunxiong Bao, Zhangjun Hu, Xianjie Liu, Libor Kobera, Sabina Abbrent, Jiri Brus, Yizheng Jin, Mats Fahlman, Richard H. Friend, Caterina Ducati, Xiao-Ke Liu, Feng Gao

**Affiliations:** 1 Department of Physics, Chemistry, and Biology (IFM), Linköping University, Linköping, 58183 Sweden; 2 Department of Materials Science & Metallurgy, University of Cambridge, 27 Charles Babbage Road, Cambridge, CB3 0FS UK; 3 Cavendish Laboratory, University of Cambridge, J J Thomson Avenue, Cambridge, CB3 0HE UK; 4 Centre for Chemistry of High-Performance & Novel Materials, State Key Laboratory of Silicon Materials, School of Materials Science and Engineering, Zhejiang University, Hangzhou, 310027 China; 5 Laboratory of Organic Electronics, Department of Science and Technology, Linköping University, Norrköping, 60174 Sweden; 6 Institute of Macromolecular Chemistry of the Czech Academy of Sciences, Heyrovskeho nam. 2, 162 06, Prague 6, Czech Republic; 7 Centre for Chemistry of High-Performance & Novel Materials, State Key Laboratory of Silicon Materials, Department of Chemistry, Zhejiang University, Hangzhou, 310027 China

**Keywords:** Materials for devices, Applied physics

## Abstract

Although perovskite light-emitting diodes (PeLEDs) have recently experienced significant progress, there are only scattered reports of PeLEDs with both high efficiency and long operational stability, calling for additional strategies to address this challenge. Here, we develop perovskite-molecule composite thin films for efficient and stable PeLEDs. The perovskite-molecule composite thin films consist of in-situ formed high-quality perovskite nanocrystals embedded in the electron-transport molecular matrix, which controls nucleation process of perovskites, leading to PeLEDs with a peak external quantum efficiency of 17.3% and half-lifetime of approximately 100 h. In addition, we find that the device degradation mechanism at high driving voltages is different from that at low driving voltages. This work provides an effective strategy and deep understanding for achieving efficient and stable PeLEDs from both material and device perspectives.

## Abstract (web summary)

The field of perovskite light-emitting diodes witnesses rapid development in both device processing strategies and performances. Here Wang *et al*. develop high-quality perovskite-molecule composite thin films and achieve high quantum efficiency of 17.3% and half-lifetime of 100 h.

## Introduction

Metal halide perovskites have attracted significant attention for light-emitting applications, because of their excellent properties such as high photoluminescence quantum efficiency (PLQE), narrow emission bandwidth, readily tunable emission spectra ranging from ultraviolet to near-infrared, and solution processability^1–4^. Recently, rapid progress has been made in perovskite light-emitting diodes (PeLEDs), with external quantum efficiencies (EQEs) reaching more than 20% through suppression of non-radiative recombination and improvement of light out-coupling efficiency^5–9^. In addition to high efficiency, it is also important to achieve PeLEDs with long operational stability, a key factor towards practical applications. However, improving operational stability of PeLEDs is still a major challenge, and obtaining PeLEDs with both high efficiency and long operational stability remains elusive.

From the materials point of view, a highly luminescent and stable perovskite film is essential to achieve PeLEDs with both high efficiency and long operational stability. Several approaches have been attempted towards this direction, including mixed A-site cations^10–14^, all-inorganic perovskites^15^, perovskite–polymer bulk heterostructures^7^, quasi-core/shell structure^6^, etc. Unfortunately, most of these approaches lead to either low efficiency or poor device stability. Not until very recently did rational additive engineering enable PeLEDs with both high efficiency and long operational stability in a few reports^5,8^.

Herein, we develop an effective approach, i.e., perovskite-molecule composite (PMC) thin films, for efficient and stable PeLEDs. Our PMC films consist of formamidinium lead iodide (FAPbI_3_) perovskite nanocrystal islands embedded in an electron-transport molecular matrix of 4,4′-diaminodiphenyl sulfone (DDS). We find that DDS controls nucleation process of the perovskite nanocrystals, leading to the formation of PMC thin films with enhanced PLQEs and prolonged PL lifetime. Moreover, this PMC structure shows impressive structural stability of α-phase FAPbI_3_ for over half a year in ambient air with relative humidity of 20–80%. By employing the PMC thin films as emissive layers, we achieve LEDs with a peak EQE of 17.3% and half-lifetime (*T*_50_, the time it takes until the light output reaches 50% of the maximum output) of 100 h at a constant current density of 20 mA cm^–2^ (initial radiance of 15 W m^−2^ sr^−1^). Considering the huge library of molecular semiconductors developed in organic LEDs^16,17^, more efficient and stable PMC thin films can be expected for achieving high-performance PeLEDs. In addition, from the device point of view, we find that the degradation mechanism of PeLEDs at high driving voltages is different from that at low driving voltages. Our findings in both materials and devices will spur further developments of PeLEDs with high efficiency and long operational stability for practical applications in lighting and display.

## Results

### PMC thin films

**Fig. 1:**
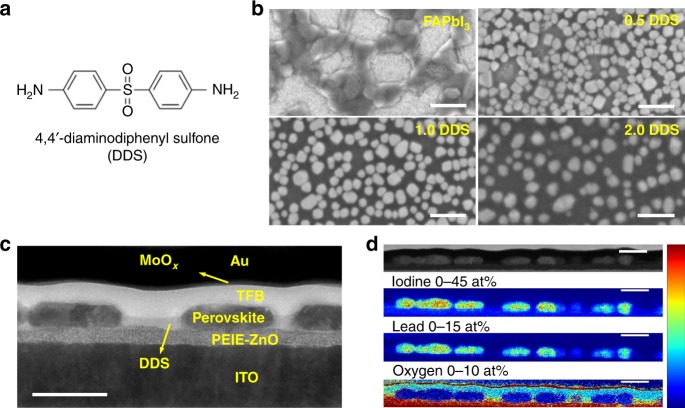
Morphology of PMC thin films. **a** Molecular structure of DDS. **b** Top-view SEM images of perovskite films. The scale bars are 500 nm. **c** Cross-sectional bright-field TEM image, and **d** cross-sectional HAADF-STEM image and associated EDS maps of the 1.0 DDS film-based LED. The scale bars are 200 nm.

The PMC thin films are prepared by facile one-step spin-coating from precursor solutions of formamidinium iodide (FAI), lead iodide (PbI_2_), and DDS dissolved in N,N-dimethylformamide (DMF) (*x* DDS films, where *x* = 0.5, 1.0 and 2.0, denote the films prepared with a DDS:Pb^2+^ molar ratio of *x*:1.0; see the “Methods” section for details). Diphenyl sulfone is widely used as an electron-transport building block in molecular semiconductors^18^, and its derivatives such as di(4-(4-diphenylaminophenyl) phenyl) sulfone have been reported as efficient blue emissive molecules^17^. In this study, a diphenyl sulfone derivative, DDS (the chemical structure shown in Fig. 1a), is chosen as the target molecular semiconductor due to its high singlet and triplet energies of 3.59 and 2.95 eV^19^, respectively, avoiding energy transfer from the perovskite nanocrystals to the molecule. In addition, DDS retains the electron-transport property of diphenyl sulfone (Supplementary Fig. 1 and Supplementary Note 1), while its amino group can help to control the growth of perovskite nanocrystals through chemical bonding with the perovskite, as will be discussed later.

The morphology of the PMC thin films is investigated with scanning electron microscope (SEM). As shown in Fig. 1b, the control film (FAPbI_3_) without DDS is a reticular film composed of irregularly shaped particles. The bright areas in this SEM image are attributed to the parts of the polyethylenimine ethoxylated-modified zinc oxide nanoparticles (PEIE-ZnO) layer which are not covered by FAPbI_3_. With addition of DDS, the perovskite particles adopt a cuboid shape. In contrast to the control film, the perovskite grains in the PMC thin films are unambiguously bright while the uncovered areas become dark with the addition of DDS. In addition, increasing amount of DDS leads to decreasing areal density of the perovskite grains.

In order to understand the composition of the areas which are not covered by the perovskite grains in the PMC thin films, we perform cross-sectional transmission electron microscopy (TEM) characterizations. We take the 1.0 DDS film as an example, which shows the best efficiency and stability among all our devices. Figure 1c is a bright-field TEM image which shows the device structure of an LED based on the 1.0 DDS film. From the bottom to the top, the layers are indium tin oxide (ITO)/PEIE-ZnO (approximately 50 nm)/emissive layer (approximately 70 nm)/poly(9,9-dioctyl-fluorene-co-N-(4-butylphenyl) diphenylamine) (TFB; approximately 60 nm)/molybdenum oxide (MoO_*x*_; approximately 7 nm)/gold (Au; approximately 60 nm). This image confirms the formation of isolated perovskite grains (dark areas) in the emissive layer. In addition, we can see that the perovskite grains are connected by gray belts whose brightness level is clearly distinct from the surrounding layers. The contrast in this bright field image is dominated by mass contrast as the cross-sectional lamella has a nominally uniform thickness, and hence regions composed of high-Z elements appear darker as they scatter more electrons^20^. Therefore, we can infer that the gray belts do not have the same composition as TFB, PEIE-ZnO, or perovskite based on their different brightness levels.

We then switch our TEM to scanning mode (STEM) to run an energy-dispersive X-ray spectroscopy (EDS) analysis. Figure 1d shows an image of the EDS scan area acquired in high-angle annular dark field mode and elemental maps of iodine, lead, and oxygen. Oxygen is selected as a marker for DDS. The iodine and lead maps show that neither element appears between perovskite grains, proving that the gray belts are not FAI, PbI_2_ or other iodine- or lead-containing compounds. The oxygen map unambiguously confirms that DDS is present on top of the PEIE-ZnO layer, right between neighboring perovskite grains. At this stage, we can conclude that the PMC thin films assemble in situ as a composite of FAPbI_3_ islands surrounded by DDS.

The cathodoluminescence (CL) spectroscopic measurements (Supplementary Fig. 2) are also consistent with the fact that DDS lies between perovskite grains in the PMC thin films. CL mapping of the 1.0 DDS film demonstrates uniform spatial distribution of emission from the perovskite grains. The CL spectrum (inset of Supplementary Fig. 2b) of a perovskite grain (Point 1) peaks at 785 nm which can be assigned to the emission of α-phase FAPbI_3_ perovskite^21^. In contrast, the dark area between perovskite grains (Point 2) shows no emission in the range of 600 to 1000 nm, indicating that there is no perovskite retained in the areas between perovskite grains.

We also note that the perovskite grains grow thicker and become more egg-like in shape with increasing amount of DDS (Supplementary Fig. 3), in good agreement with the top-view SEM images in Fig. 1b. Such morphology may contribute to improved EQE in devices as a result of enhanced light out-coupling^5^.

### Properties of PMC thin films

**Fig. 2:**
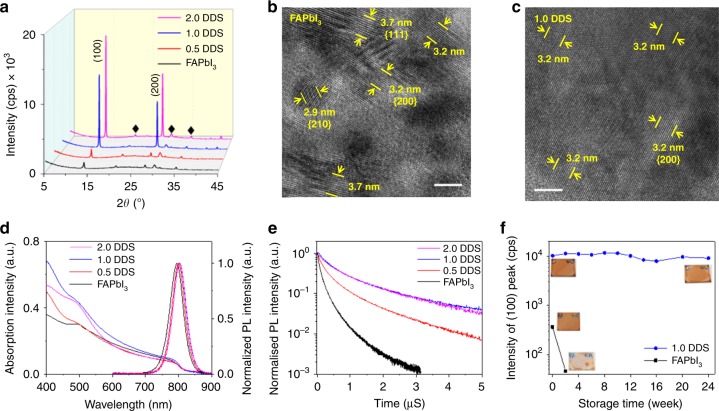
Properties of PMC thin films. **a** XRD patterns of perovskite films; diffraction peaks from the ITO substrate are marked as ♦. Cross-sectional HRTEM images of (**b**) the FAPbI_3_ control film and (**c**) the 1.0 DDS film. The measured lattice spacings (10 fringes for each pair of markers) match well with the cubic α-phase FAPbI_3_ structure. The scale bars for (**b**) and (**c**) are 5 nm. **d** Absorption and PL spectra and **e** time-correlated single-photon counting spectra (recorded at a fluence of 0.13 μJ cm^−2^) of perovskite films. **f** XRD intensity of the (100) diffraction peak of perovskite films (inset: photographs of the fresh and aged perovskite films).

In order to examine the quality of PMC thin films, we investigate the crystal structure, optical properties, and storage stability. Figure 2a shows X-ray diffraction (XRD) patterns of all four samples. They display peaks at 13.9° and 27.9° which are assigned to (100) and (200) planes of α-phase FAPbI_3_ perovskite, respectively^22,23^. The low intensities of these peaks in the FAPbI_3_ control film and 0.5 DDS samples indicate their poor crystallinity. In contrast, the intensities of both peaks rise by more than one order of magnitude in the 1.0 DDS and 2.0 DDS samples (Supplementary Table 1), demonstrating vastly improved crystallinity. The full width at half maximum (FWHM) values of these peaks decrease with increasing DDS content, indicating larger perovskite grain size. The intense and sharp diffraction peaks of the 1.0 DDS and 2.0 DDS films demonstrate their high crystallinity even though there are large amounts of organic molecules in these films. In addition, Grazing-incidence wide-angle x-ray scattering (GIWAXS) patterns (Supplementary Fig. 4 and Supplementary Note 2) further demonstrate enhanced long-range-orientated crystallization along both out-of-plane and in-plane directions in the PMC thin films. These results suggest high crystallinity 3D perovskite nanocrystals in the 1.0 DDS and 2.0 DDS films.

High crystallization quality in the 1.0 DDS film is further confirmed by high-resolution TEM (HRTEM) image of the perovskite grains. As shown in Fig. 2b, the presence of Moiré fringes and many differently orientated lattice fringes confirm that the FAPbI_3_ control film is polycrystalline with differently orientated grains. The measured lattice spacings match well with the cubic α-phase FAPbI_3_ structure^23^. In contrast, the HRTEM image of the 1.0 DDS film (Fig. 2c) shows a uniform set of lattice fringes with *d*-spacing value of 3.2 Å across the field of view. This matches the lattice spacing of {200} planes in α-phase FAPbI_3_. Furthermore, a lower-magnification HRTEM image with a larger field of view (48 nm × 48 nm) also shows uniform lattice fringes and fast Fourier transform (FFT) patterns across the entire image (Supplementary Fig. 5). This suggests that the perovskite grains in the 1.0 DDS film can be nanoscale single crystals.

Note that we do not observe any diffraction peak of 2D phases in the XRD patterns of PMC thin films (Fig. 2a). This is also confirmed by absorption and PL spectroscopy. The absorption spectra of the PMC thin films (Fig. 2d) show features similar to that of the 3D FAPbI_3_ control film, but with a slightly decreased bandgap (Supplementary Fig. 6), possibly due to different dielectric environment in different films. Moreover, the PMC thin films show narrower PL spectra with increasing amount of DDS (FWHM of FAPbI_3_: 94 meV; 0.5 DDS film: 88 meV; 1.0 DDS and 2.0 DDS films: 86 meV), indicating improved structure ordering of the perovskite nanocrystals in the PMC thin films^5^.

We use the time-correlated single-photon counting (TCSPC) technique to investigate charge-carrier kinetics of these films. As shown in Fig. 2e, the FAPbI_3_ control film shows a short PL lifetime. The addition of DDS significantly prolongs the PL lifetimes of the PMC thin films. Meanwhile, the PMC thin films show enhanced PLQEs in a large range of excitation power (Supplementary Fig. 7). The prolonged PL lifetimes and enhanced PLQEs demonstrate reduced trap density of perovskite nanocrystals in these PMC thin films.

In addition, the PMC thin films demonstrate excellent long-term stability under the exposure to moisture and oxygen. As shown in Fig. 2f and Supplementary Fig. 8a, the fresh FAPbI_3_ control film shows relatively weak diffraction intensity of the (100) peak at 13.9˚ which disappears after 2-week storage in ambient air with humidity of 20 to 80%. Meanwhile, a new peak at 11.7˚ appears, indicating that the α-phase FAPbI_3_ in the control film completely transforms into the δ-phase during storage^24^. Concomitantly, the color of the FAPbI_3_ control film changes from dark brown to yellow as shown in the insets of Fig. 2f. In contrast, the color of the 1.0 DDS film does not change after storage in ambient air for half a year. The 1.0 DDS film maintains pure α-phase FAPbI_3_ without decomposition or phase transformation; no diffraction peak of either PbI_2_ or δ-phase FAPbI_3_ is observed (Supplementary Fig. 8b) and the intensity of the (100) diffraction peak remains unchanged after storage for 24 weeks in ambient air (Fig. 2f). These results demonstrate excellent structural stability of the PMC thin films against moisture and oxygen in ambient air.

### The roles of the molecule DDS in PMC thin films

The excellent optical properties and structural stability of the PMC thin films motivate us to investigate the crystal growth mechanism of these films and the role(s) of DDS.

**Fig. 3:**
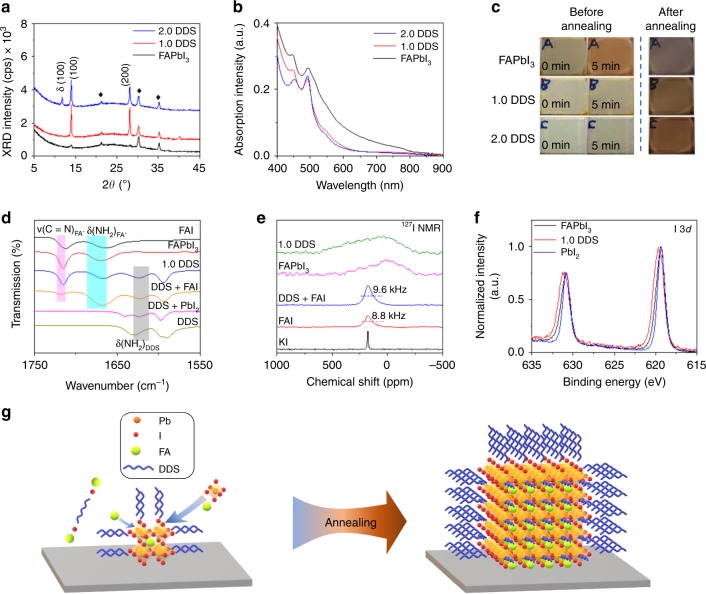
Crystal growth mechanism of PMC thin films. **a** XRD patterns (diffraction peaks from the ITO substrate are marked as ♦) and **b** absorption spectra of perovskite films before annealing. **c** The photographs of perovskite films before and after annealing. **d** FTIR spectra of annealed FAPbI_3_, 1.0 DDS films, and their raw materials. **e** The experimental ^127^I NMR spectra of KI, FAI, FAI + DDS, FAPbI_3_, and 1.0 DDS films. **f** X-ray photoelectron spectroscopy (XPS) of I 3d peak of annealed PbI_2_, FAPbI_3_, and 1.0 DDS films. **g** Schematic illustration of the growth mechanism of PMC thin films.

All of the films before annealing show diffraction peaks at 13.9° and 27.9° in the XRD patterns (Fig. 3a), indicating the formation of α-phase FAPbI_3_ already before annealing, possibly due to the presence of excess FAI in the precursor^25,26^. The diffraction intensities of the FAPbI_3_ control film is much weaker than those of the PMC thin films. In spite of stronger diffraction intensities in the XRD measurements, the PMC thin films show much weaker absorption (Fig. 3b) in the wavelength range of 600 to 800 nm. The difference in the absorption of these films before annealing can also be clearly visualized by the photographs shown in Fig. 3c. The color of the FAPbI_3_ control films quickly changes from yellow to orange within 5 min after the spin-coating process; in contrast, the color of the 1.0 DDS films remains yellow with high transparency within 5 min after spin-coating, and the 2.0 DDS films are completely colorless. These combined XRD and absorption measurements suggest that the addition of DDS leads to the formation of a few amount of yet high-quality α-FAPbI_3_ crystal nuclei after spin-coating. In other words, one role of DDS is to retard perovskite nucleation process, which assists the formation of high-quality perovskite films^27,28^. Annealed with extended durations, the 1.0 DDS films exhibit increased diffraction intensity, larger grain size with similar shape, and lower areal density of perovskite nanocrystals (Supplementary Fig. 9).

With all these results, we can now conclude that during the deposition of the PMC thin films, high-quality α-phase FAPbI_3_ crystal nuclei form with the existence of DDS, continuously growing into large crystals during annealing. At this stage, it remains unclear how DDS facilitates the formation of high-quality α-phase FAPbI_3_ crystal nuclei and how DDS interacts with the perovskite lattices after annealing.

In order to answer these questions, we perform attenuated total reflectance-Fourier transform infrared (ATR-FT-IR) spectroscopy to elucidate how DDS works with the perovskites. Firstly, we examine the interactions between DDS and FAI. As shown in Fig. 3d, the NH_2_ scissoring vibration δ(NH_2_)_DDS_ of DDS at around 1630 cm^–1^ shifts to lower wavenumber (1623 cm^–1^) in the DDS + FAI mixture film (prepared from a DMF solution with DDS:FAI = 1:2.2 in molar ratio). This indicates that the N–H chemical bond of the –NH_2_ groups in DDS is weakened with the addition of FAI, suggesting that there is hydrogen bonding between the –NH_2_ groups in DDS and FAI. This hydrogen bonding could in principle come from the interaction between –NH_2_ and FA^+^ or between –NH_2_ and I^–^, the former of which can be excluded based on the following evidence. The formation of hydrogen bond (if any) between the C=NH_2_^+^ group in FAI and –NH_2_ in DDS is expected to weaken the C=N and N–H chemical bonds, leading to shifts of C=N stretching vibration ν(C=N)_FA_ and δ(NH_2_)_FA_ to lower wavenumbers^8,29^. However, both ν(C=N)_FA_ and δ(NH_2_)_FA_ of FA^+^ ions shift to higher wavenumbers in the DDS + FAI mixture film, suggesting that there is no hydrogen bond forming between FA^+^ and DDS. We notice similar shifts to high wavenumbers when mixing FAI with PbI_2_ to form perovskites, where the C=N and N–H chemical bonds of the FA^+^ ions are strengthened. In that case, I^–^ anions contribute to the formation of corner-sharing [PbI_6_]^4–^ octahedra, hence decrease the interaction between I^–^ anions and FA^+^.

In order to provide direct evidence for the interaction between DDS and I^–^, ^127^I nuclear magnetic resonance (NMR) spectroscopy measurements were conducted (Fig. 3e). Relatively sharp signals of FAI and FAI + DDS are found at ca. 200 ppm. The significant change in signal half-width of FAI + DDS (9.6 kHz) in comparison to signal half-width of neat FAI (8.8 kHz) confirms interactions between DSS and I^–^. Similar effect is observed in the case of neat FAPbI_3_ and FAPbI_3_ + DDS (1.0 DDS) systems. The clear broadening of the detected signal indicates a weak interaction of iodine anions. This observed asymmetric broadening can further indicate the formation of different chemical entities (different clusters) in local structures.

We also examine the interactions between DDS and PbI_2_. We notice the split of the δ(NH_2_)_DDS_ peak in the DDS + PbI_2_ mixture film (Fig. 3d), indicating that there are chemical interactions between the –NH_2_ groups in DDS and Pb^2+^ or between the –NH_2_ groups and I^–^. In order to further understand these interactions, we perform X-ray photoelectron spectroscopy (XPS) measurements. As shown in Supplementary Fig. 10, the Pb 4f spectra of the FAPbI_3_ and 1.0 DDS films keep the same compared with that of the PbI_2_ film. However, the I 3d spectrum of the FAPbI_3_ film is broader (Fig. 3f) compared to that of the PbI_2_ film, suggesting the change of chemical environment of I^–^ anions in the FAPbI_3_ film. In addition, the I 3d spectrum of the 1.0 DDS film becomes broader and shifts to higher binding energies compared to the FAPbI_3_ control film. This observation proves that the change of chemical environment of I^–^ anions is not only influenced by the formation of the perovskite phase but also the existence of DDS.

By combining above FTIR, NMR, and XPS results, we conclude that the –NH_2_ groups in DDS form hydrogen bonds with I^–^ anions, rather than Pb^2+^ or FA^+^ cations. Supplementary Fig. 11 show the electropositivity nature of the –NH_2_ groups in DDS, further rationalizing the interaction between the –NH_2_ groups in DDS and I^–^ anions as discussed in Supplementary Note 3.

Figure 3g is a schematic illustration that shows the growth mechanism of PMC thin films and the roles of DDS. The –NH_2_ groups of DDS form coordination bonds with I^–^ ions, consequently retarding the perovskite nuclei process. Upon annealing, FAI contributes to the formation of the perovskite lattices and the DDS-FAI interaction breaks. Instead, the –NH_2_ groups of DDS form coordination bonds with the I^–^ ions at the surface of the perovskite crystals. Supplementary Fig. 12 shows further evidences that the vibrations of FA^+^ ions vary before and after annealing while the vibrations of –NH_2_ groups of DDS show little difference.

We note that the growth mechanism of the PMC thin films and the roles of DDS agree well with their excellent optical properties. The retarded crystal formation process caused by DDS leads to slow growth of high-quality α-phase FAPbI_3_ crystals; the DDS molecules, wrapping at the surface of perovskite crystals, could prevent water/oxygen molecules from penetrating into the perovskite lattices, leading to long-term structural stability of the PMC thin films in ambient conditions.

### LED devices based on PMC thin films

**Fig. 4:**
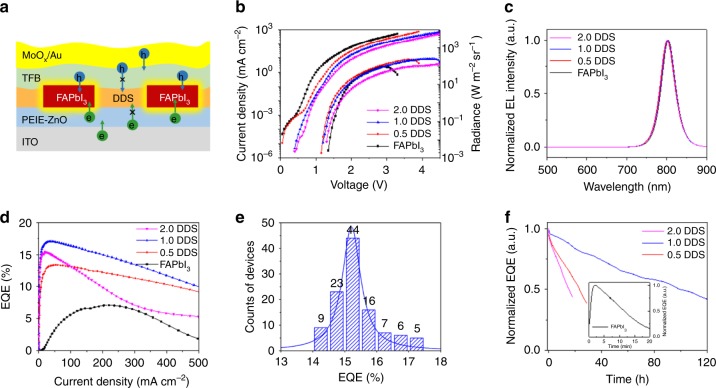
Characteristics of PMC thin film-based LEDs. **a** Schematic illustration of the PMC thin film-based LEDs. **b** Current density-voltage-radiance curves of LEDs. **c** Normalized EL spectra of LEDs at 2.0 V driving voltage. **d** EQE-current density curves of the LEDs. **e** Histogram of peak EQEs of 110 LEDs based on the 1.0 DDS films. **f** Operational stability of PMC thin films-based LEDs (instet: FAPbI_3_-based LED) at a constant current density of 20 mA cm^−2^.

Encouraged by the excellent optical properties and crystal structural stability of the PMC thin films, we investigate the device performances by fabricating LEDs using the PMC thin films as emissive layers. The device structure of the LEDs is shown in Fig. 4a and verified by TEM in Fig. 1c. Current density-voltage-radiance characteristics of these devices are shown in Fig. 4b. The control device based on the FAPbI_3_ control film shows large current density of 1.01 mA cm^−2^ at 1.0 V. In contrast, the 1.0 DDS film-based LED shows a far lower current density of 0.02 mA cm^–2^ at 1.0 V. In addition, the 1.0 DDS film-based LED shows low current density of 10^−3^ to 10^−5^ mA cm^–2^ in the voltage range of –0.5 to 0 V (Supplementary Fig. 13). This proves that the addition of DDS greatly suppresses leakage current. The EL spectra of these devices are almost identical, peaking at 802 nm (Fig. 4c). However, the EQEs are dependent on the emissive layers. As a result of improved quality of the perovskite nanocrystals in the PMC thin films, the devices based on PMC thin films show remarkably enhanced EQEs (Fig. 4d). The control device shows a peak EQE of 7.0% at a current density of 203.1 mA cm^−2^ (2.6 V), whereas the device based on the 1.0 DDS film shows the highest EQE of 17.3% at a current density of 28 mA cm^−2^ (2.2 V). In addition, the device based on the 1.0 DDS film exhibits low efficiency roll-off with J_50%_ (current density at which the EQE drops to half of its maximum value)^30^ of up to 620 mA cm^−2^. The histogram of EQEs for 110 devices shows an average EQE of 15.4%, with a relative standard deviation of 4.5% (Fig. 4e).

In addition, the LEDs based on the PMC thin films exhibit notably longer operational stability. As shown in Fig. 4f, the control device based on the FAPbI_3_ film shows a short *T*_50_ of 11.6 min at a constant current density of 20 mA cm^−2^ with an initial radiance of 0.9 W m^−2^ sr^−1^. In contrast, devices based on the 0.5 DDS, 1.0 DDS, and 2.0 DDS films exhibit notably improved *T*_50_ values of 24, 100, and 16 h, respectively, at a constant current density of 20 mA cm^−2^ with an initial radiance of 12 W m^−2^ sr^−1^, 16 W m^−2^ sr^−1^, and 15 W m^−2^ sr^−1^, respectively. We note that LEDs based on the 1.0 DDS film exhibit the best balance between efficiency and operational stability among the reported PeLEDs (Supplementary Table 3), demonstrating the advantage of the PMC thin films in fabricating PeLEDs with both high efficiency and long operational stability. Considering the huge library of molecular semiconductors developed in organic LEDs^16,17^, this work will bridge perovskites and semiconducting molecules, and spur further development of PMC films, opening up more routes for achieving efficient and stable perovskite LEDs.

## Discussion

**Fig. 5:**
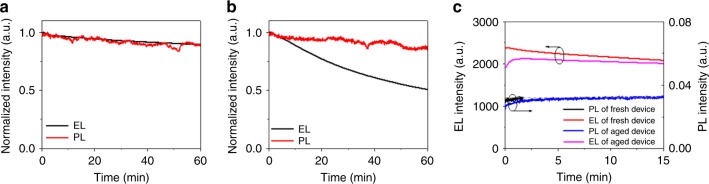
PL and EL intensities of a working 1.0 DDS film-based LED. Simultaneously measured PL and EL intensities at (**a**) a low driving voltage of 2.1 V and (**b**) high driving voltage of 2.8 V, respectively. **c** Alternate EL and PL intensity measurements of a device before and after aging (Black line: first measure the PL intensity on the fresh device; red line: turn off the laser and measure the EL intensities at a constant current density of 100 mA cm^−2^ for 15 min; blue line: remove bias and measure the PL intensities again; magenta line: turn off the laser and measure the EL intensities again at a constant current density of 100 mA cm^−2^). The EL intensity measurements at 100 mA cm^−2^ are the aging processes.

We also measure the operational stability of the 1.0 DDS film-based LED at various constant current densities. As shown in Supplementary Fig. 14, the lifetime drops dramatically with increasing current densities. In addition to the emissive layer, many other factors such as the stability of the charge-transport layers, the location of recombination zone, etc., may affect the stability (operational lifetime and efficiency roll-off) of an LED^16,31,32^. To obtain a deep understanding of the stability of the PMC thin film-based LEDs, we simultaneously measure PL and EL decay characteristics of a working LED based on the 1.0 DDS film^33^. As shown in Fig. 5a, at a relatively low driving voltage of 2.1 V, the device shows a similar decay trend for both EL and PL, suggesting that the degradation of the device is mainly caused by the emissive layer. In contrast, the EL intensity of the device decreases much faster than the PL intensity at a relatively high driving voltage of 2.8 V (Fig. 5b).

In order to understand this phenomenon, we perform alternate PL and EL intensity measurements on the same 1.0 DDS film-based LED. As shown in Fig. 5c, after electrical aging (at a constant current density of 100 mA cm^–2^ for 15 min, red line), the PL intensities (blue line) are almost identical to those of fresh device (black line), which means that the short-time aged PMC emissive layer can almost recover to its initial state. This result is consistent with the observation in the transient PL decay measurement (Supplementary Fig. 15 and Supplementary Note 4). However, the EL intensities of aged device (magenta line) are lower than those of fresh device (red line), suggesting the formation of EL quenchers. Note that the EL quenchers do not influence the PL intensity, which may be explained by the degradation of the interfaces between the emissive layer and charge-transport layers^34^. In addition, we find obvious emission from TFB (400 to 500 nm) when the driving voltage is above 2.5 V (Supplementary Fig. 16), which may be induced by carrier leakage and/or charge imbalance at high driving voltages^32^. These results suggest that the degradation of the interface between the emissive layer and charge-transport layer could be one of the main channels for the degradation of the whole 1.0 DDS film-based LED at high driving voltages. We thus suggest that in addition to the perovskite material, the degradation of the interfaces between the emissive layer and charge-transport layers should be paid special attention.

In conclusion, we have developed PMC thin films, which consist of in situ formed high-quality perovskite nanocrystals embedded in the electron-transport molecular matrix, for efficient and stable PeLEDs. The molecular matrix controls the nucleation process of the perovskite nanocrystals, and significantly improves the PLQEs and long-term stability under the exposure to moisture and oxygen. Consequently, PeLEDs based on the PMC thin films reach a peak EQE of 17.3% and half-lifetime of 100 h at a constant current density of 20 mA cm^−2^ (initial radiance of 15 W m^−2^ sr^−1^). In addition, we find that the degradation of our PeLEDs at a relatively low driving voltage is mainly caused by the degradation of the emissive layer; whereas at a relatively high driving voltage the degradation of the interfaces between the emissive layer and charge-transport layers could be one of the main degradation channels. The findings in this work from both material and device perspectives pave the way for achieving efficient and stable PeLEDs.

## Methods

### Materials

Colloidal ZnO nanocrystals were synthesized following the reported solution-precipitation process^3^. Formamidinium iodide (FAI) was obtained from Dyesol, and PbI_2_ was obtained from TCL (≥ 98.0% purity). Other chemicals were obtained from Sigma-Aldrich.

### PMC precursor solutions

The PMC precursor solutions were prepared by dissolving DDS, FAI and PbI_2_ in N,N-dimethylformamide (DMF, Sigma-Aldrich, anhydrous, 99.8%) solution. Take 1.0 DDS precursor solution as an example: the 1.0 DDS precursor solution was prepared by dissolving DDS, FAI and PbI_2_ with a molar ratio of 1:2.2:1 in DMF (the concentration of Pb^2+^ is 0.1 M) and then the solution was put on a hot plate and stirred at 60 ^o^C overnight. The other PMC precursor solutions with different amounts of DDS were prepared by the same process but with different amounts of DDS. The reference FAPbI_3_ perovskite precursor solution was prepared by dissolving FAI and PbI_2_ with a molar ratio of 2.2:1 in DMF solution (the concentration of Pb^2+^ is 0.1 M).

### Film preparation

ITO-coated glasses were soaked in a solution mixed with deionized water, ammonium hydroxide (25%) and hydrogen peroxide (28%) (5:1:1 by volume) and then heated at 100 °C for 15 min. The substrates were then cleaned with deionized water and dried by nitrogen flow. Colloidal ZnO nanocrystals were deposited on ITO at 4000 rpm for 30 s in air. Then polyethylenimine ethoxylated (PEIE) in isopropyl alcohol (0.03 wt%) was spin-coated onto ZnO at 5000 rpm for 30 s and then annealed at 100 °C for 10 min in the glovebox. The PMC precursor solutions were spin-coated onto PEIE-ZnO films at 4000 rpm for 30 s, followed by thermal annealing at 100 ^o^C for 5 min.

### Device fabrication

Based on the film preparation procedures, LEDs were completed with the following processes: TFB in chlorobenzene (12 mg mL^−1^) was spin-coated at 3000 rpm for 30 s. Then the films were transferred to a vacuum chamber, in which MoO_*x*_ and Au were deposited at the rates of 0.2 Å s^−1^ and 1.5 Å s^−1^, respectively, at vacuum of 1 × 10^−6^ Torr.

### Device characterizations

Characterization of the LED was carried out at room temperature in a nitrogen-filled glovebox. Current density-voltage (J-V) characteristics were recorded by Keithley 2400 source meter with a step of 0.05 V. Forward-viewing spectral radiant flux was measured by an integrating sphere coupled with a QE65 Pro spectrometer. The active device area was 0.0725 cm^2^.

### Operational stability measurement

The operational stability of the devices (without encapsulation) was measured in a testing box (shown in Supplementary Fig. 14c) (stored in the N_2_-filled glovebox) driven by D3000-16 system, with HMC 8100 providing a constant current. A photodetector tests the feedback photoelectric current.

### Film characterizations

XRD patterns were obtained from an X-ray diffractometer (Pananalytical X’Pert Pro) with an X-ray tube (Cu Kα, *λ* = 1.5406 Å). Steady-state PL spectra of the films were recorded by using a 450 nm laser as an excitation source. Absorption spectra were measured with a PerkinElmer model Lambda 900. Scanning electron microscopy (SEM, Philips XL30 FEG SEM) was operated at 3 keV to characterize the morphology of the samples.

### CL spectroscopy measurement

CL spectroscopy measurements were performed using a monoCL4 system combined with a LEO 1550 Gemini scanning electron microscope (SEM). A fast CCD detection system and a Peltier cooled photomultiplier tube have been used for acquisition of CL spectra and for CL mapping, respectively.

### TEM measurement

Cross-sectional sample lamellae were cut and thinned to electron transparency (~200 nm) with a FEI Helios Nanolab Dualbeam FIB/SEM following a standard protocol^35^. The lamellae were then directly transferred into a FEI Osiris TEM operated at 200 kV, minimising air exposure to about 2 min. STEM-EDS SIs were acquired using a defocused beam (Δ*f* = –1 μm) with a beam current of approximately 250 pA, dwell time of 50 ms per pixel, and spatial sampling of 10 nm per pixel. STEM-EDS SIs were analysed in HyperSpy with principal component analysis for denoising^36^. Bright field and HRTEM images were acquired at areas which have not been previously exposed to the electron beam. Images were taken quickly (seconds) using low-dose imaging conditions.

### GIWAXS measurement

All the synchrotron radiation-based GIWAXS measurements were performed at BL14B1 beamline, Shanghai Synchrotron Radiation Facility (SSRF). The diffraction patterns were collected by two dimensional MarCCD 225 detector with a distance of 234 mm from samples to the detector. All the samples were protected with N_2_ gas during the measurements. To assure the diffraction intensity, an exposure time of 15 s was adopted with an incidence angle of 0.2°, and the wavelength of the X-ray was 1.24 Å (10 KeV).

### Attenuated total reflectance-Fourier transform infrared

The ATR-FT-IR spectra were recorded from a PIKE MIRacle ATR accessory with a diamond prim in a Vertex 70 Spectrometer (Bruker) using a DLaTGS detector at room temperature. The spectra were acquired at 2 cm^–1^ resolution.

### X-ray photoelectron spectroscopy

XPS tests were carried out using a Scienta ESCA 200 spectrometer in ultrahigh vacuum (approximately 1 × 10^–10^ mbar) with a monochromatic Al (K alpha) X-ray source providing photons with 1486.6 eV. The XPS experimental condition was set so that the FWHM of the clean Au 4*f*_7/2_ line (at the binding energy of 84.00 eV) was 0.65 eV. All spectra were measured at a photoelectron take-off angle of 0° (normal emission).

### Time-correlated single-photon counting (TCSPC) measurement

TCSPC was performed with a home-built setup, which contains diode lasers driven by a DH400, PicoQuant laser controller, a monoch/romator coupled with a micro channel plate photomultiplier tube (from Hamamatsu-R3809U-50) and TCSPC electronics (Lifespec-pc and VTC900 PC card from Edinburgh Instruments). A 407 nm laser generates pulses with FWHM of 80 ps and repetition rates of 20 kHz.

### NMR measurement

High-resolution NMR measurements were recorded on a Bruker 600 MHz Avance III spectrometer at Larmor frequencies ν(^127^I) = 120.111 MHz. For ^127^I NMR measurements with 90° pulse of 14 μs, relaxation delay 10 s, 1024 scans were used, and ^127^I spectra were collected as a one piece-spectrum at an offset equal to +12 kHz. The signal was calibrated with 0.01 M KI in D_2_O, set at 0.0 ppm. During detection of the ^127^I NMR experiments WALTZ65 decoupling sequence was used to eliminate heteronuclear spin-spin coupling. The temperature was kept constant at 298 ± 0.2 K with a BVT 3000 temperature unit during measurements. All samples were dissolved in DMF at room temperature in inert atmosphere.

### Simultaneous measurements of PL and EL intensity

The simultaneous measurements of PL and EL intensities were obtained by a homemade setup consisting of a lock-in amplifier (SR830), an electric meter (Keithley 2000, Keithley 2400), and a photodetector (Thorlabs PDA100A)^33^. The devices were encapsulated by glasses with ultraviolet-curable glue. A continuous-wave 450 nm laser was used as the light source, together with an optical chopper for frequency modulation (80 Hz). The intensity of the laser light was kept at a low level for low PL intensity (PL/EL < 10^–4^) to reduce the influence from photon-generated charge-carriers. The EL and PL signals can be separated by using the lock-in amplifier.

## Supplementary information

Supplementary Information

## Data Availability

The datasets generated and/or analyzed during the current study are available from the corresponding author on reasonable request.
