# Supplementary material for: Perovskite-molecule composite thin films for efficient and stable light-emitting diodes
Source: Nat Commun. 2020 Feb 14;11:891. doi: 10.1038/s41467-020-14747-6 (PMC7021679; doi:10.1038/s41467-020-14747-6)
Supplement: Supplementary file 1 — Supplementary Information [file 41467_2020_14747_MOESM1_ESM.pdf]

Supplementary Information

Perovskite-molecule composite thin films for efficient and stable light-emitting diodes

Wang et al

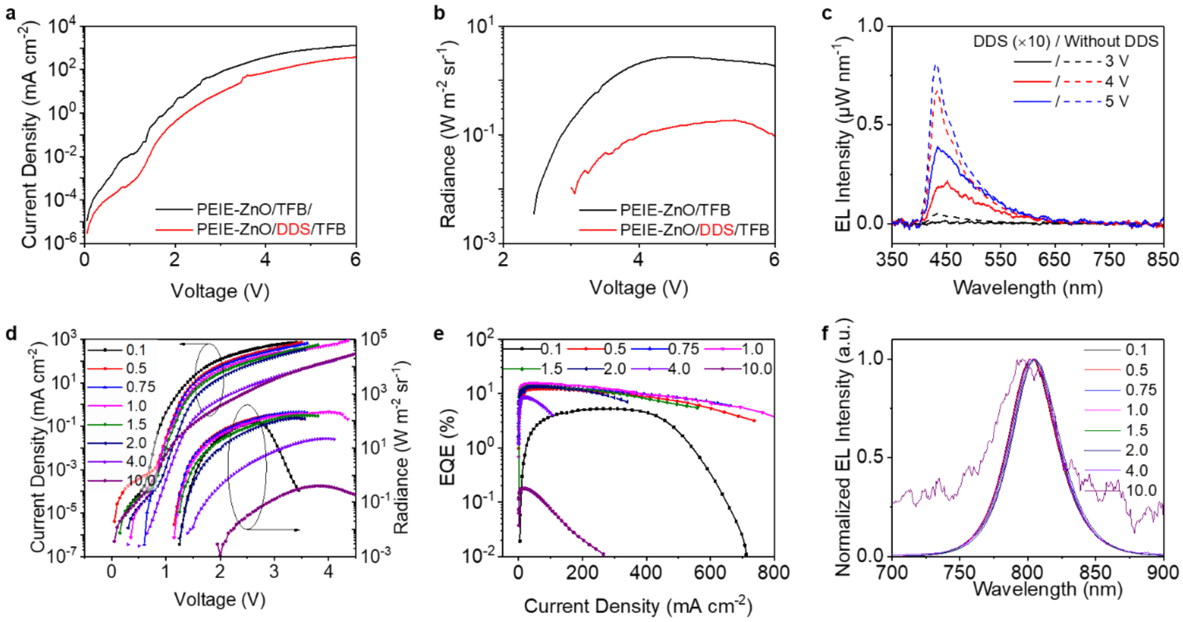

Supplementary Figure 1. (a) Current density-voltage, (b) radiance-voltage curves and (c) EL spectra at different driving voltages of devices with configurations of ITO/ PEIE-ZnO/ TFB/ MoO_x/ Au and ITO/ PEIE-ZnO/ DDS/ TFB/ MoO_x/ Au. (d) Current density-voltage-radiance curves, (e) EQE-current density curves and (f) normalized EL spectra of PMC thin films-based LEDs with different amounts of DDS ($FAPbI_3$:DDS = 0.1, 0.5, 0.75, 1.0, 1.5, 2.0, 4.0, 10.0).

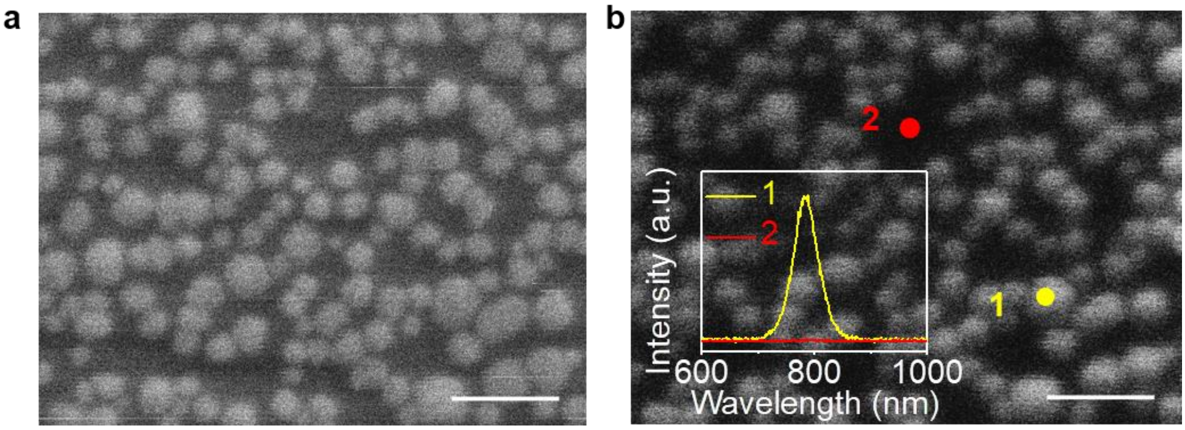

Supplementary Figure 2. (a) Top-view SEM image for cathodoluminescence (CL) spectroscopy. (b) Cathodoluminescence (CL) mapping and spectra (inset) of the 1.0 DDS film. The scale bars are 500 nm.

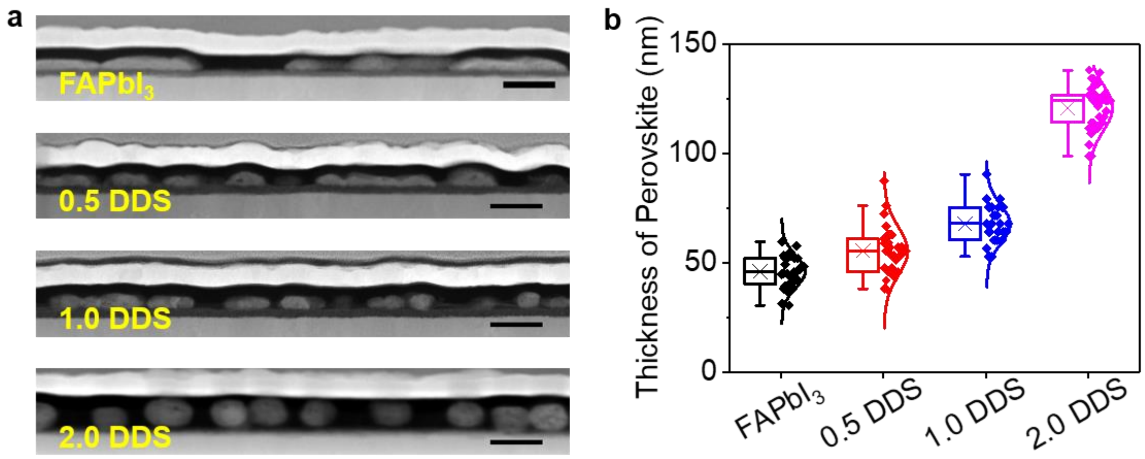

Supplementary Figure 3. (a) Cross-sectional HAADF-STEM images of PeLEDs. The scale bars are 250 nm. (b) The thickness of perovskite layers measured at 30 points for each PeLED.

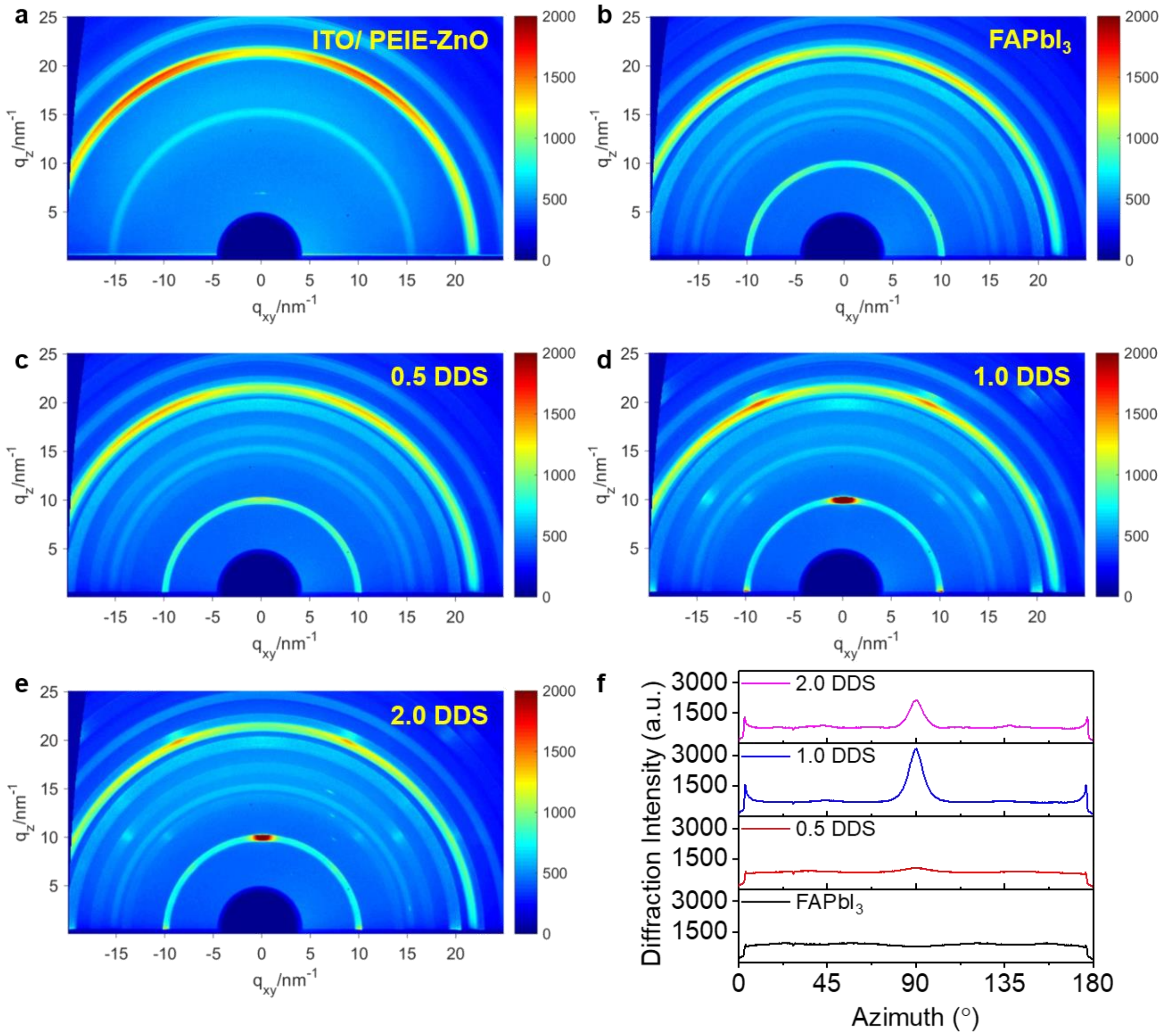

Supplementary Figure 4. GIWAXS patterns of (a) ITO/ PEIE-ZnO substrate, (b-e) $FAPbI_3$, 0.5 DDS, 1.0 DDS, 2.0 DDS perovskite films prepared on ITO/ PEIE-ZnO substrate. (f) Integration along the azimuthal directions q_{xy} = 10 nm^{-1}.

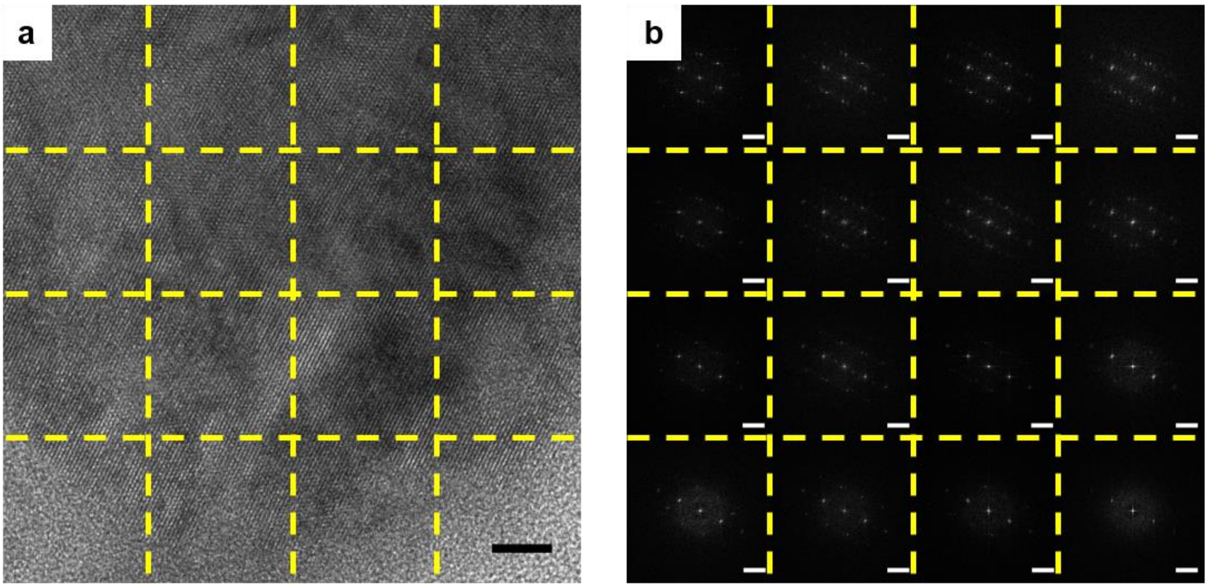

Supplementary Figure 5. (a) Cross-sectional HRTEM image of 1.0 DDS film divided into 16 regions of interest (ROIs). The scale bar is 5 nm. (b) Fast Fourier transformation (FFT) patterns taken from each ROI and arranged in the same order as the ROIs. The scale bars are 3 nm^{-1}.

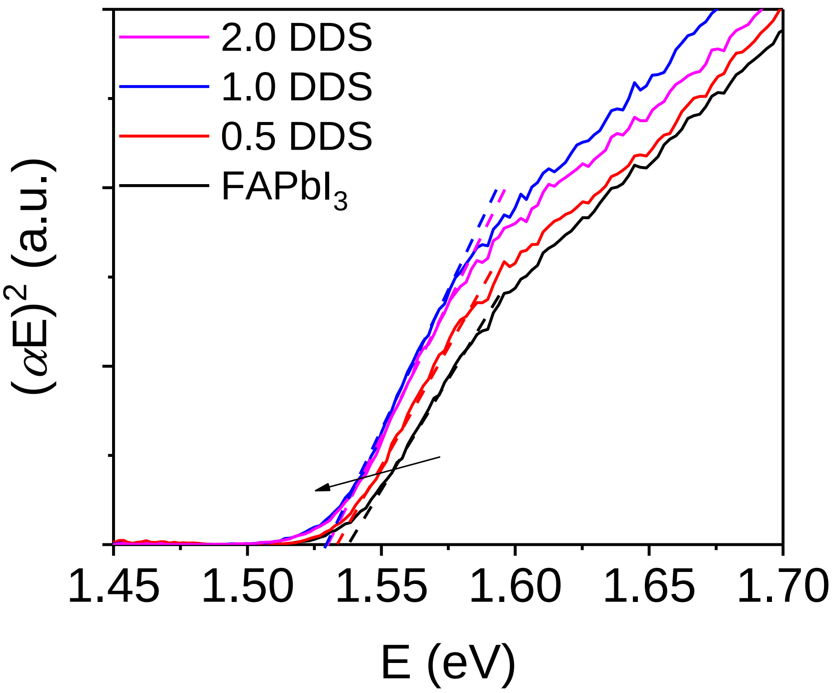

Supplementary Figure 6. The $(\alpha E)^2$–energy plot of perovskite films.

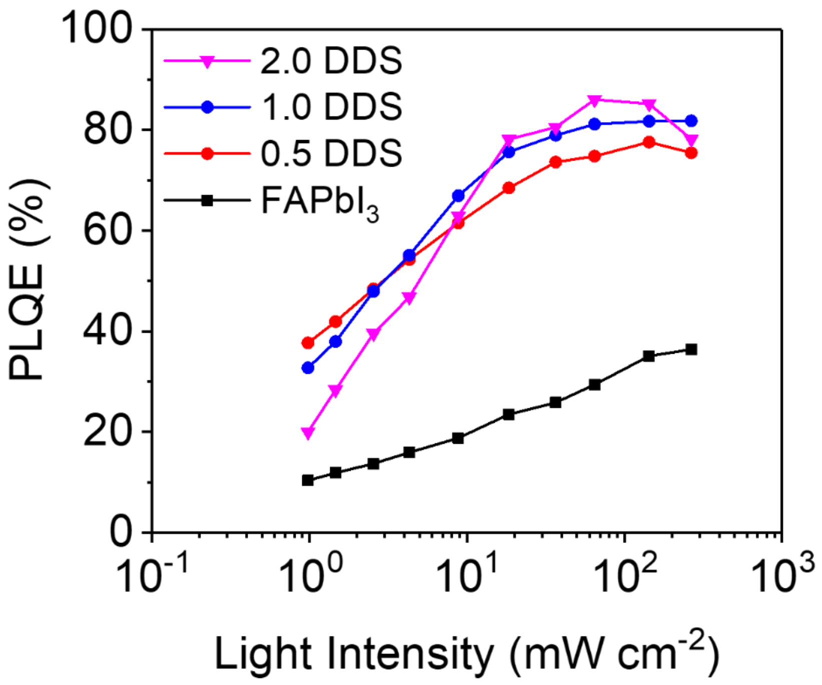

Supplementary Figure 7. Power-dependent photoluminescence quantum efficiencies (PLQEs) of perovskite films.

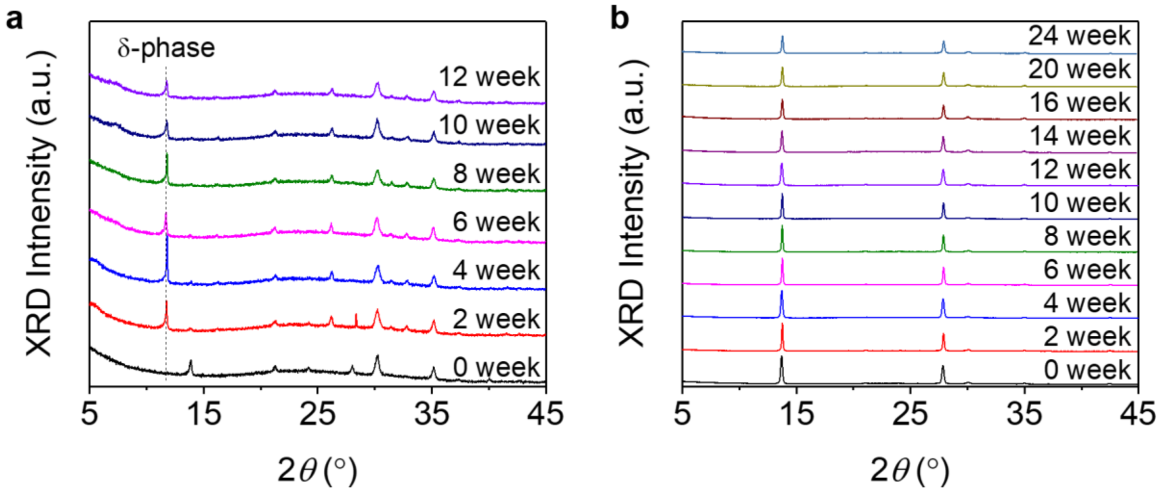

Supplementary Figure 8. XRD patterns of perovskite films with different storage time in ambient air. (a) $FAPbI_3$ perovskite film. (b) 1.0 DDS film.

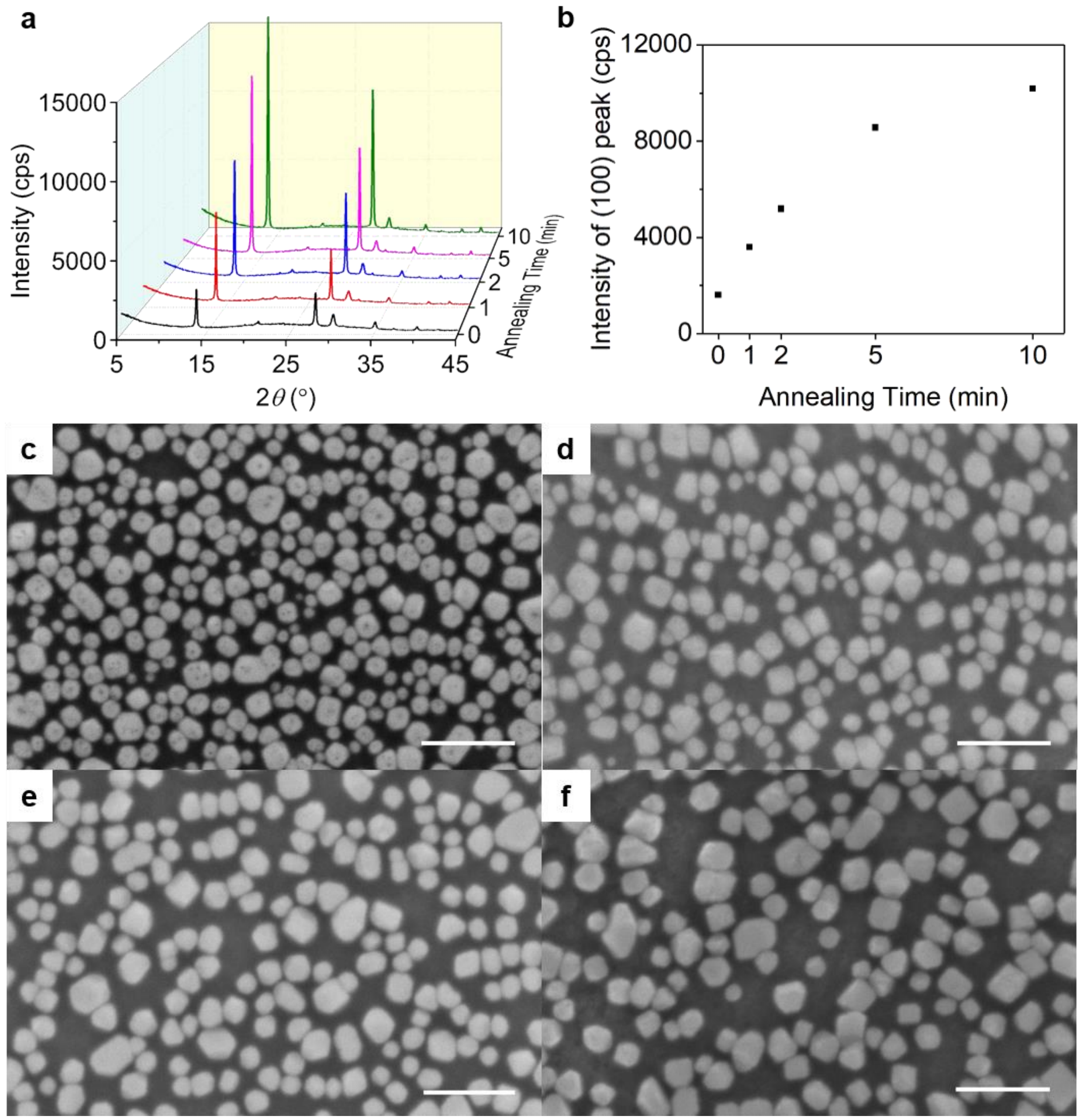

Supplementary Figure 9. (a) XRD patterns and (b) intensity of the (100) peak of 1.0 DDS films with different annealed extended durations. Top-view SEM images of 1.0 DDS films annealed for (c) 1 min, (d) 2 min, (e) 5 min and (f) 10 min. The scale bars are 500 nm.

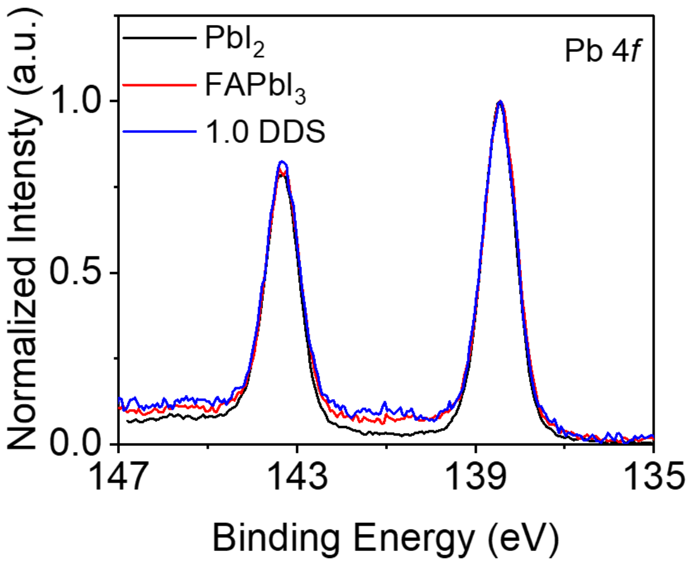

Supplementary Figure 10. The X-ray photoelectron spectroscopy (XPS) of Pb 4*f* spectra of PbI_2, $FAPbI_3$, and 1.0 DDS films.

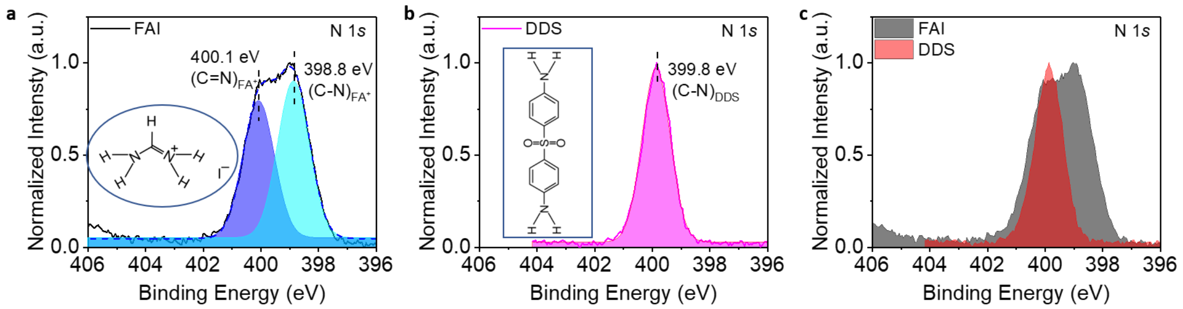

Supplementary Figure 11. The X-ray photoelectron spectroscopy (XPS) of N 1*s* spectra of (a) FAI film and (b) DDS film, with their molecular structures shown in the insets. (c) N 1*s* spectra of FAI and DDS films.

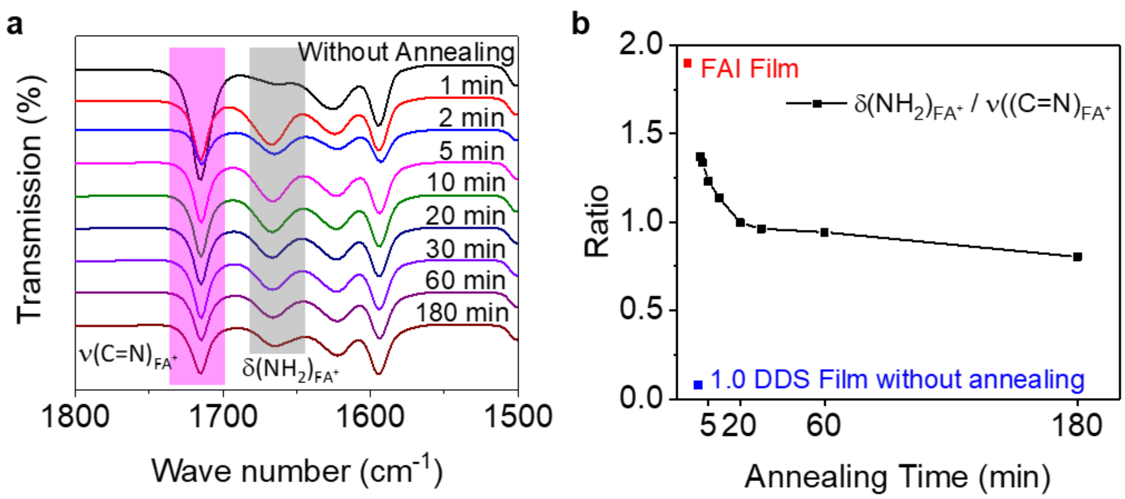

Supplementary Figure 12. (a) FTIR spectra and (b) ratios between δ(NH_2) and ν(C–N) of FA^+ of the 1.0 DDS film with different annealing time. The red and blue dots in (b) represent δ(NH_2)/ν(C–N) ratios of the pure FAI film and unannealed 1.0 DDS film, respectively.

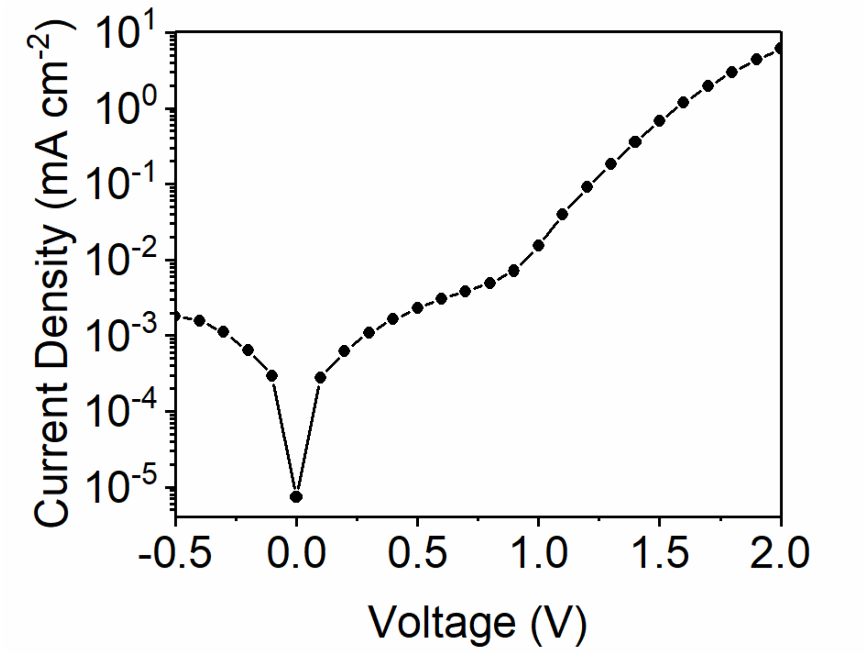

Supplementary Figure 13. The current density-voltage curve of 1.0 DDS film-based LED in the range of –0.5 to 2 V.

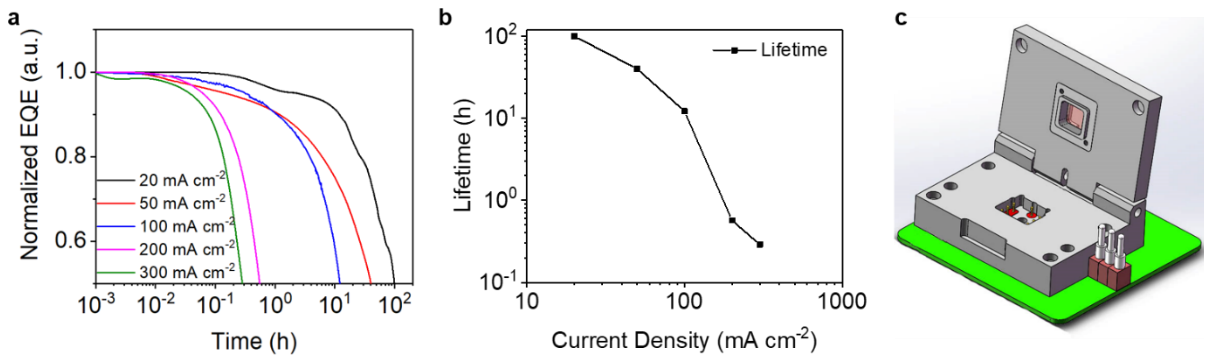

Supplementary Figure 14. (a-b) Operational stability of LEDs at different constant current densities. (c) The testing box for stability measurements.

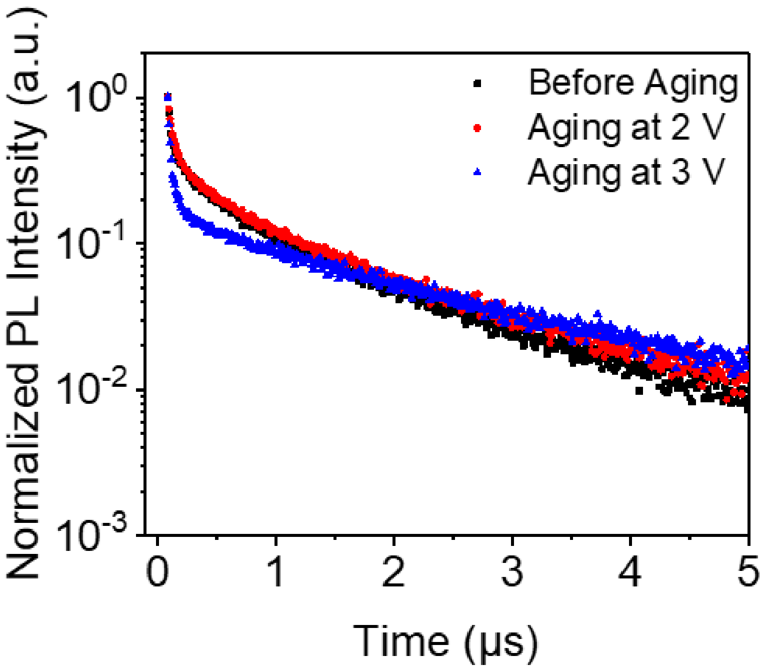

Supplementary Figure 15. Transient PL decay curves of 1.0 DDS film-based LED before and after electrical aging at 2 V and 3 V for 15 min, respectively.

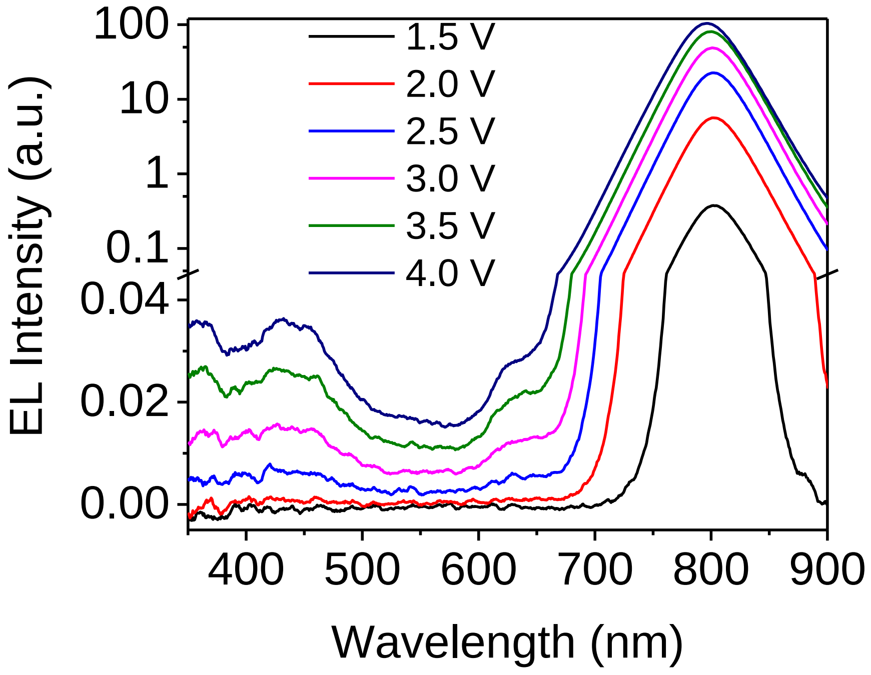

Supplementary Figure 16. EL spectra of the LED based on 1.0 DDS film at different driving voltages.

Supplementary Table 1. XRD characteristics of (100) peak of perovskite films.

Film	Intensity (cps)	FWHM (^{o})
$FAPbI_3$	885	0.2746
0.5 DDS	1157	0.1506
1.0 DDS	10946	0.1004
2.0 DDS	12578	0.0836

Supplementary Table 2. Summary of the representative stability of PeLEDs.

Perovskite material	EL Peak (nm)	EQE (%)	Measurement condition	Lifetime	Ref.
$BA_2MA_4Pb_5I_{16}$	744	0.5	2 V	14 h (T_{50})	1
$NFPI_6B_2$	763	11.7	10 mA cm^{-2}	1 h (T_{50})	2
$MAPbI_3$: BAI	748	10.4	3 mA cm^{-2}	5 h (T_{75})	3
$MAPbI_3$	749	7.9	3 mA cm^{-2}	10 h (T_{90})	4
NFACsPbI	783	7.8	10 mA cm^{-2}	15 h (T_{50})	5
(Cs/MA/FA)PbBr/I	569	7.3	3 mA cm^{-2}	7.9 h (T_{50})	6
$CsPbBr_3$	518	10.5	100 cd m^{-2}	250 h (T_{50})	7
(Cs/MA)$PbBr_3$	525	20.31	166.67 mA cm^{-2}	10.42 min (T_{50})	8
$FAPbI_3$	803	20.7	100 mA cm^{-2}	20 h (T_{50})	9
CsPbBr/I	653	21.3	~100 cd m^{-2}	3 h (T_{50})	10
$FAPbI_3$	800	21.7	25 mA cm^{-2}	20 h (T_{50})	11
Perovskite–polymer bulk heterostructure	~795	20.1	0.1 mA cm^{-2}	45 h (T_{50})	12
**PMC thin film**	**802**	**17.3**	**20 mA cm^{-2}**	**100 h (T_{50})**	**This work**

Supplementary Note 1

As shown in Supplementary Fig. 1a-c, the device with DDS works well as an LED with TFB as the emissive layer and hole-transport layer, and DDS and PEIE-ZnO as electron-transport bilayers. Both of them show emission from the TFB layer.[13] It indicates that DDS can serve as an electron-transport layer. This could be similar when DDS is added into the perovskite films. The PMC thin films-based LEDs with larger amounts of DDS (over 100% in the molar ratio compared to $FAPbI_3$) show relatively lower current densities but still work well as shown in Supplementary Fig. 1d-f, further confirming the electron-transport property of DDS.

Supplementary Note 2

In order to confirm that the PMC films show improved textures, we perform GIWAXS measurements. All samples (Supplementary Fig. 4b-e) show diffraction patterns at the same q_{xy} positions indicating the same phase they bear. For the control $FAPbI_3$ film, the dominant diffraction ring at q_{xy} = 10 nm^{-1}, corresponding to the (100) peak of α-phase $FAPbI_3$, shows an almost constant diffraction intensity along the azimuth angle. With addition of DDS and increasing amount from 0% to 50%, 100% and 200%, a clear diffraction mottling occurs at the dominant diffraction ring at q_{xy} = 10 nm^{-1}, implying stronger diffraction intensities. To analyse the evolution of microstructural arrangement, we integrate the GIWAXS patterns azimuthally over the ring at q_{xy} = 10 nm^{-1}. As depicted in Supplementary Fig. 4f, the control film shows an almost equally distributed diffraction intensity along the azimuth angle from 0° to 180°. After loading of 50% DDS (0.5 DDS film), a slightly preferred orientation occurs at the azimuth angle of 90°. Further increasing the amount of DDS to 100% and 200% (1.0 DDS and 2.0 DDS films), the orientation along the azimuth angle of 90° becomes the dominant preferred crystal orientation accompanied with two peaks at the azimuth angle of 0° and 180°. In the context of crystal microstructure, the changes of the diffraction mottling indicate an enhanced long-range-orientated crystallization along both out-of-plane and in-plane directions.

Supplementary Note 3

We note that the –NH_2 group of commonly used alkyl amines is usually a Lewis base, which is supposed to coordinate with cations (Lewis acids) rather than I^- anions (a Lewis base). However, we find that DDS is an aryl amine and its –NH_2 groups are electropositive in nature, which is confirmed by the N 1s spectra of FAI and DDS. As shown in Supplementary Fig. 11a, FAI has two different –NH_2 groups, C=NH_2^+ and C–NH_2, showing two distinct N 1s peaks at 400.1 and 398.8 eV, respectively. This finding is consistent with the report.[14] However, DDS shows only one N 1s peak at 399.8 eV (Supplementary Fig. 11b). Interestingly, this peak is closer to the higher binding energy C=NH_2^+ peak of FAI (Supplementary Fig. 11c), indicating that the –NH_2 groups of DDS have similar chemical environment (electropositivity) compared to that of the C=NH_2^+ group of FAI. The electropositivity of the –NH_2 groups of DDS can be attributed to the electron-drawing nature of –(C_6H_4)SO_2(C_6H_4)– group.[15]

Supplementary Note 4

As shown in Supplementary Fig 15, we measured transient PL of 1.0 DDS film-based LED before and after aging at 2 V and 3 V for 15 min, respectively. The device after electrical aging at 2 V shows the same PL decay as that before electrical aging. It indicates that the perovskite layer has not been seriously damaged after short time aging at a relatively low voltage. This result agrees with the EL & PL intensity measurements of 1.0 DDS film-based device in Fig. 5a, which does not show obvious degradation after 15 min. In contrast, the device after electrical aging at 3 V shows a shorter fast decay channel and a longer slow decay channel. These PL decay measurements, combined with the EL & PL intensity measurements in Fig. 5b and c, lead us to believe that the change of PL decay is due to the degradation of interfaces between the emissive layer and charge-transport layers.

Supplementary References

1 Tsai, H., *et al.* Stable light-emitting diodes using phase-pure ruddlesden-popper layered perovskites. *Adv Mater* **30**, 1704217, (2018).

2 Wang, N., *et al.* Perovskite light-emitting diodes based on solution-processed self-organized multiple quantum wells. *Nat Photonics* **10**, 699-704, (2016).

3 Xiao, Z., *et al.* Efficient perovskite light-emitting diodes featuring nanometre-sized crystallites. *Nat Photonics* **11**, 108-115, (2017).

4 Song, L., *et al.* Efficient inorganic perovskite light-emitting diodes with polyethylene glycol passivated ultrathin $CsPbBr_3$ films. *J Phys Chem Lett* **8**, 4148-4154, (2017).

5 Yang, M., *et al.* Reduced efficiency roll-off and enhanced stability in perovskite light-emitting diodes with multiple quantum wells. *J Phys Chem Lett* **9**, 2038-2042, (2018).

6 Kim, H. P., *et al.* High-efficiency, blue, green, and near-infrared light-emitting diodes based on triple cation perovskite. *Adv Opt Mater* **5**, 1600920, (2017).

7 Wang, H., *et al.* Trifluoroacetate induced small-grained $CsPbBr_3$ perovskite films result in efficient and stable light-emitting devices. *Nat Commun* **10**, 665, (2019).

8 Lin, K., *et al.* Perovskite light-emitting diodes with external quantum efficiency exceeding 20 per cent. *Nature* **562**, 245-248, (2018).

9 Cao, Y., *et al.* Perovskite light-emitting diodes based on spontaneously formed submicrometre-scale structures. *Nature* **562**, 249-253, (2018).

10 Chiba, T., *et al.* Anion-exchange red perovskite quantum dots with ammonium iodine salts for highly efficient light-emitting devices. *Nat Photonics* **12**, 681-687, (2018).

11 Xu, W., *et al.* Rational molecular passivation for high-performance perovskite light-emitting diodes. *Nat Photonics* **13**, 418-424, (2019).

12 Zhao, B., *et al.* High-efficiency perovskite–polymer bulk heterostructure light-emitting diodes. *Nat Photonics* **12**, 783-789, (2018).

13 Jitsui, Y., Ohtani, N. Transmission electron microscope observation of organic–inorganic hybrid thin active layers of light-emitting diodes. *Nanoscale Res Lett* **7**, 591, (2012).

14 Chen, P., *et al.* In situ growth of 2D perovskite capping layer for stable and efficient perovskite solar cells. *Adv Funct Mater* **28**, (2018).

15 Liu, Y., Li, C., Ren, Z., Yan, S., Bryce, M. R. All-organic thermally activated delayed fluorescence materials for organic light-emitting diodes. *Nat Rev Mater* **3**, (2018).
